# Coming home to die? The association between migration and mortality in rural Tanzania before and after ART scale-up

**DOI:** 10.3402/gha.v7.22956

**Published:** 2014-05-21

**Authors:** Francis Levira, Jim Todd, Honorati Masanja

**Affiliations:** 1 Data Analysis Cluster Unit, Ifakara Health Institute, Dar es Salaam, Tanzania; 2 Department of Population Health, London School of Hygiene and Tropical Medicine, London, UK

**Keywords:** residents, internal migration, external migration, rural-urban migrants, mortality, HIV, ART, ARV, Tanzania

## Abstract

**Background:**

Prior to the scale-up of antiretroviral therapy (ART), demographic surveillance cohort studies showed higher mortality among migrants than residents in many rural areas.

**Objectives:**

This study quantifies the overall and AIDS-specific mortality between migrants and residents prior to ART, during ART scale-up, and after widespread availability of ART in Rufiji district in Tanzania.

**Design:**

In Health and Demographic Surveillance System (HDSS), the follow-up of individuals aged 15–59 years was categorized into three periods: before ART (1998–2003), during ART scale-up (2004–2007), and after widespread availability of ART (2008–2011). Residents were those who never migrated within and beyond HDSS, internal migrants were those who moved within the HDSS, and external migrants were those who moved into the HDSS from outside. Mortality rates were estimated from deaths and person-years of observations calculated in each time period. Hazard ratios were estimated to compare mortality between migrants and residents. AIDS deaths were identified from verbal autopsy, and the odds ratio of dying from AIDS between migrants and residents was estimated using the multivariate logistic regression model.

**Results:**

Internal and external migrants experienced higher overall mortality than residents before the introduction of ART. After widespread availability of ART overall mortality were similar for internal and external migrants. These overall mortality experiences observed were similar for males and females. In the multivariate logistic regression model, adjusting for age, sex, education, and social economic status, internal migrants had similar likelihood of dying from AIDS as residents (adjusted odds ratio [AOR]=1.14, 95% confidence interval [CI]: 0.70–1.87) while external migrants were 70% more likely to die from AIDS compared to residents prior to the introduction of ART (AOR=1.70, 95% CI: 1.06–2.73). After widespread availability of ART with the same adjustment factors, the odds of dying from AIDS were similar for internal migrants and residents (AOR=1.56, 95% CI: 0.80–3.04) and external migrants and residents (AOR=1.42, 95% CI: 0.76–2.66).

**Conclusions:**

Availability of ART has reduced the number of HIV-infected migrants who would otherwise return home to die. This has reduced the burden on rural communities who had cared for the return external migrants.

Rural-urban migration pattern is the dominant form of mobility in developing countries characterized by youth migrating to urban cities in search for work, better life, and advanced education (1). The reverse pattern is commonly characterized by people returning home after retirement (2–4). A substantial proportion of research on migrations and health has focused much on the growing population of urban migrants, where emphasis has been on increased vulnerability to poor health and acquisition of diseases relative to urban residents. However, upon their return to rural areas, little is known about their health status and survival relative to rural residents. Public health research on migration patterns provides insights on factors that drive mobility and their influence on health-seeking behavior, disease transition, and mortality.

In the process of integrating with urban residents, rural-urban migrants are more likely to be exposed to HIV-related risky sexual behavior and HIV infection than their urban counterpart (5–7). In developing countries, there is a growing evidence of higher HIV prevalence for return rural migrants compared to rural residents (8–10). In South Africa, Welaga described the mortality differentials between migrants and nonmigrants in a rural community in South Africa (11). After adjusting for age, sex, and social economic status, this study showed higher overall mortality and elevated odds of dying from AIDS for external migrants compared to residents. Another rural cohort study in a similar setting in South Africa reported higher mortality for external migrants than residents in communities with circular labor migrants (12).

Before the introduction of free antiretroviral therapy (ART), the majority of infected migrants could not afford treatment cost in urban centers approximated at US$35/month, and often returned to their rural home villages to seek care from traditional healers, and to be looked after by their families in anticipation of death (13–17). In Thailand, Knodel reported consistent patterns of adults with AIDS migrating home to seek care from their parents at final stages of illness (17).

Overall ART coverage in Tanzania was estimated at 42% among adults and 18% among children by 2010, following the introduction of free ART services (18–21). Several studies have investigated the differentials in treatment access and reported higher loss to follow-up in the continuum of care, less good adherence to care, and treatment by migrants than residents (22–25). We anticipate and hypothesize that the scaling-up of free ART program may possibly contribute to a reduction in return migrants seeking terminal care in rural areas (26–30). However, there are few reports on mortality among rural migrants in Africa, and how this has been affected by HIV and the availability of ART.

Evaluating differentials in mortality among migrants and residents in rural requires continuous follow-up and documentation of death events of both migrants and residence. In addition, evaluation of AIDS-related mortality requires further follow-up that documents cause of death for both migrants and residence. This study focuses on the longitudinal follow-up of residents and migrants from 1998 to 2011 in a rural setting in Rufiji district in Tanzania. The district is located 170 km from Dar-es-Salaam, the largest and closest city with approximately 4.36 million inhabitants. Majority of migrants from Rufiji relocate to Dar-es-salaam while maintaining links with their parents and families. This study quantifies differences in overall and AIDS-specific mortality between migrants and residents and investigates changes in the mortality risks and likelihood of dying from AIDS before ART was introduced, during ART scale-up, and after widespread availability of ART in the rural district of Rufiji in Tanzania.

## Methods

### Study area

This study used data collected from Rufiji Health and Demographic Surveillance System (HDSS) in Tanzania. The HDSS covers 38 villages, with approximately 95,000 individuals in about 19,000 households and it covers approximately 50% of district population. Rufiji HDSS is a member of the INDEPTH Network (31) and of the Analysing Longitudinal Population-based HIV data in Africa (ALPHA) network (32).

The surveillance is designed as an open cohort of individuals who enter through initial census enumeration, birth, and in-migration and exit through out-migration, lost to follow-up, and death. An initial census was conducted between October 1998 and January 1999 by enumerating all individuals in the selected study area. Basic demographic events of birth, deaths, in- and out-migration were regularly documented during household visits that are normally conducted once in every 4 months. Baseline socioeconomic (education and occupation) and environmental (source of drinking water and type of toilet facilities) factors were collected during the initial census and in follow-up rounds.

### Residency status definition

**Fig. 1:**
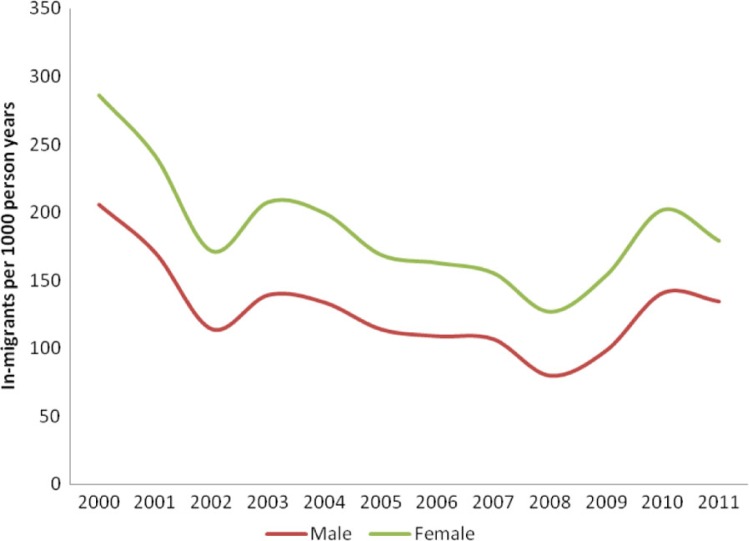
Trends in in-migration for males and females (2000–2011).

Individuals migrating into the surveillance area are registered if they have been in the surveillance for at least 4 months and are expecting to stay in the study area for a longer period of time, or if they are married to a resident. Information on reasons for migration and where the migrant came from is also collected. Annually about 6,000 individuals aged 15–59 years migrate within or into the study area. Patterns of migration for males and females between 2000 and 2011 are shown in Fig. 1.

*Residents* were defined as all individuals who were present during initial census, or were born into the cohort, or who had resided in the same household for 2.5 years. Individuals who migrated into the study area from outside the surveillance area were classified as *external migrants* from the date of migration until 2.5 years after migration. Those who changed location within the study area were considered as *internal migrants* from the date of migration until 2.5 years after migration. After 2.5 years, both external and internal migrants were considered *residents*.

### ART period definition

All individuals aged 15 to 59 years in the study area from 2000 to 2011 were included in the analysis. The total person-years of observation (PYO) were calculated from 1 January 2000, or the date the individual entered the cohort, at the age of 15 years, or through migration until the date the person died, left the HDSS, was lost to follow-up, attained the age of 60 years, or at the end of analysis period (31 December 2011). The observation time was divided into three periods for this analysis: period prior to availability of ART (2000–2003), period when ART was scaled-up (2004–2007), and period of widespread availability of ART (2008–2011). Within each period, all deaths and person-years of follow-up were recorded, and the overall mortality rates were calculated for residents and migrants separately.

### Cause of death

The cause of death information of all registered deaths was derived from verbal autopsy (33). The process involves interviewing close relatives of the deceased through a structured questionnaire that details an account of the sequence of events and symptoms leading to death. Two sets of questionnaire were implemented between 1999 and 2011, but the basic questions necessary to detect AIDS deaths did not differ between the two questionnaires. Structured questionnaires were then reviewed by two independent physicians and the cause of death was assigned using International Classification of Diseases, tenth revision (ICD10) (34). A third physician was involved in cases of disagreement between the two. Where we have all three discordant, the cause was registered as ‘undetermined’. Validated studies have shown that the verbal autopsy procedure is among reliable tools for identifying AIDS deaths in the absence of reliable medical death certificate (35–37).

### Statistical analysis

Descriptive analysis described all cause and AIDS-related mortality for migrants and residents stratified by sex and ART period. Predictive analysis used statistical models to compare residents and migrants adjusted for sex, age, level of education, and social economic status. Age categories were generated by splitting residency episodes at age 15, 25, 40, and 60 years. Education level was collected during regular updates of household and individual level variables. Social economic status variable was generated from principal component analysis of household ownership of selected items including bicycle, car, motor cycle, radio, fridge, TV, watch, land, sofa set, bed, video, mattress, water pump, animals such as cow, tailoring machine, satellite dish, iron, fan, concrete floor, water sources, and type of energy used for cooking (38).

### Multivariate survival model

Since Rufiji HDSS does not collect data on HIV status of participants, all cause mortality rates were estimated for residents and external and internal migrants with mortality rate ratios (RRs) and 95% confidence intervals (95% CI) comparing migrants to residents. In each ART period, we compared all cause mortality for residents and migrants using multivariate Poisson regression obtaining hazard rate ratios (HRR) and 95% CIs, adjusting for possible confounders.

### Logistic regression for AIDS deaths

We evaluated AIDS mortality from the verbal autopsy cause of death, defining a binary response variable of AIDS death or not AIDS death. In each ART period, we used a multivariate logistic regression to assess the adjusted odds ratio (AOR) of dying from AIDS for external migrants and internal migrants compared to residents, adjusting for age, sex, education, and socioeconomic status. Stata software version 12 (StataCorp, College Station, TX) was used to perform all statistical analysis.

## Results

### Overall mortality in 4 years

Table 1 shows results of longitudinal follow-up of adults aged 15–59 years with a total of 428,010 person-years, and 2,653 deaths recorded from 2000 through 2011. Crude mortality rates were stratified by gender and ART periods, and were higher for external migrants than residents before and during ART scale-up (RR=1.38, 95% CI: 1.16–1.63 and RR=1.40, 95% CI: 1.13–1.64, respectively). After widespread availability of ART, mortality was comparable for residents and external migrants (RR=1.14, 95% CI: 0.92–1.40). Similar patterns of overall mortality were observed for males and females.

**Table 1:** Adult (aged 15 to 59 years of age) deaths, person-years of observation (PYO), overall mortality and mortality RR over ART periods stratified by gender

	Before ART				ART scale-up				Widespread of ART			
					
	Deaths	PYO	Rate	RR	Deaths	PYO	Rate	RR	Deaths	PYO	Rate	RR
Overall												
Residents	629	94.9	6.6	1	577	98.9	5.8	1	558	108.9	5.1	1
Internal	194	24.0	8.1	1.2^a^	177	26.6	6.7	1.1^a^	103	22.0	4.7	0.9^a^
External	167	18.3	9.1	1.4^a^	135	17.0	7.9	1.4^a^	113	19.3	5.9	1.1^a^
Overall	990	137.3	7.2		889	142.5	6.2		774	150.2	5.2	
Male												
Residents	306	46.6	6.6	1	285	49.5	5.8	1	267	55.1	4.8	1
Internal	73	9.1	8.0	1.2^a^	55	10.2	5.4	0.9^a^	40	8.3	4.8	1.0^a^
External	76	8.0	9.5	1.4^a^	63	7.0	9.1	1.6^a^	47	8.0	5.9	1.2^a^
Males	455	63.7	7.1		403	66.7	6.0		354	71.4	5.0	
Females												
Residents	323	48.3	6.7	1	292	49.4	5.9	1	291	53.8	5.4	1
Internal	121	14.9	8.1	1.2^a^	122	16.4	7.5	1.3^a^	63	13.7	4.6	0.8^a^
External	91	10.3	8.8	1.3^a^	72	10.1	7.2	1.2^a^	66	11.3	5.8	1.0^a^
Females	535	73.6	7.3		486	75.8	6.4		420	78.8	5.3	

RR- Mortality rate ratio of external and internal migrants over residents estimated using Mantel-Haenszel method.

a Mortality rates are different (*p*<0.05).

b Mortality rates are comparable (*p*>0.05).

PYO in thousands.

Internal migrants were observed with higher mortality than residents before ART (RR=1.22, 95% CI: 1.04–1.43), while lower mortality rates comparable to residents were observed during scale-up and after widespread availability (RR=1.14, 95% CI: 0.96–1.35 and RR=0.91, 95% CI: 0.74–1.13), respectively. Sex-specific mortality rates for residents and internal migrants were comparable over all ART periods, except during ART scale-up when female internal migrants were observed with higher mortality than residents.

Estimates from the multivariate survival model in Table 2 indicate that internal and external migrants had a higher mortality rate than residents after adjusting for sex, age, education, and economic status. Compared to residents, higher mortality rates were observed among external migrants [adjusted hazard ratio (AHR)=1.54, 95% CI: 1.30–1.84] and internal migrants (AHR=1.24, 95% CI: 1.05–1.46) before ART was available. During ART scale-up, external migrants continued to show persistently higher mortality rates than residents (AHR=1.43, 95% CI: 1.17–1.73), while internal migrant's mortality was higher compared residents but not statistically significant (AHR=1.13, 95% CI: 0.95–1.35). Internal and external migrants had comparable mortality rates to residents after widespread availability of ART.

**Table 2:** Adjusted relative risk for multivariate models for the 4 years survival experience by migration status and ART period

	Before ART	ART scale-up	Widespread of ART
Migration status			
Residents	1	1	1
Internal	1.24 (1.05–1.46)	1.13 (0.95–1.35)	0.98 (0.79–1.22)
External	1.54 (1.30–1.84)	1.43 (1.17–1.73)	1.20 (0.97–1.48)
Sex			
Male	1	1	1
Female	0.99 (0.87–1.13)	0.99 (0.87–1.14)	0.95 (0.82–1.10)
Education			
Tertiary	1	1	1
Primary	1.12 (0.85–1.48)	1.28 (0.92–1.77)	1.36 (0.90–2.05)
Secondary	0.66 (0.41–1.07)	0.81 (0.50–1.32)	0.55 (0.31–0.97)
No education	0.95 (0.71–1.26)	1.16 (0.83–1.63)	1.43 (0.94–2.17)
Age			
15–25	1	1	1
25–40	2.66 (2.22–3.18)	3.13 (2.54–3.86)	2.36 (1.89–2.96)
40–59	3.66 (3.04–4.42)	4.92 (3.97–6.08)	4.26 (3.42–5.30)
Economic status			
5th Least poor	1	1	1
1st Poorest	1.01 (0.82–1.24)	1.03 (0.83–1.28)	0.99 (0.79–1.25)
2nd quintile	0.77 (0.62–0.96)	0.72 (0.57–0.91)	0.88 (0.69–1.11)
3rd quintile	0.79 (0.64–0.97)	0.81 (0.65–1.01)	0.66 (0.52–0.84)
4th quintile	0.83 (0.68–1.02)	0.93 (0.75–1.14)	0.85 (0.68–1.07)
Missing	1.78 (1.43–2.21)	2.03 (1.57–2.63)	1.07 (0.81–1.42)

Adjusted mortality risk ratio of migrants over residents estimated using parametric regression model with exponential distribution adjusted for sex, education, age and social economic status.

Confidence interval estimated at 95% significant level.

### Logistic regression model for AIDS deaths

Table 3 shows the number of deaths assessed using verbal autopsy and the proportion that was identified as AIDS deaths. In the crude analysis, there were no significant associations between AIDS death and residency status within males and females separately and over all ART periods. In a multivariate logistic regression of all deaths (Table 4), adjusting for sex, age, education, and social economic status, external migrants were 70% more likely to die from AIDS compared to residents before the introduction of ART (AOR=1.70, 95% CI: 1.06–2.73). During ART scale-up and after widespread availability of ART, there were no significant differences in the odds of AIDS mortality between residents, internal migrants, and external migrants.

**Table 3:** Characteristics of participants included in the AIDS mortality model

	Before ART		ART scale-up		Widespread of ART	
			
Variable	Deaths	AIDS deaths (%)	Deaths	AIDS deaths (%)	Deaths	AIDS deaths (%)
Overall						
Residents	620	72 (11.6)	527	71 (13.5)	461	76 (16.5)
Internal	191	27 (14.1)	163	30 (18.4)	74	16 (21.6)
External	164	32 (19.5)	127	23 (18.1)	89	18 (20.2)
Overall	**975**	**131 (13.4)**	**817**	**124 (15.2)**	**624**	**110 (17.6)**
Male						
Residents	304	36 (11.8)	263	27 (10.3)	218	24 (11.0)
Internal	73	3 (4.1)	46	4 (8.7)	32	6 (18.8)
External	75	15 (20.0)	59	9 (15.3)	36	3 (8.3)
All males	452	54 (11.9)	368	40 (10.9)	286	33 (11.5)
Female						
Residents	316	36 (11.4)	264	44 (16.7)	243	52 (21.4)
Internal	118	24 (20.3)	117	26 (22.2)	42	10 (23.8)
External	89	17 (19.1)	68	14 (20.6)	53	15 (28.3)
All females	523	77 (14.7)	449	84 (18.7)	338	77 (22.8)

These were the overall (both males and females).

**Table 4:** Adjusted odds ratio for multivariate logistic regression model for factors associated with AIDS deaths by migration status and ART period

	Before ART	ART scale-up	Widespread of ART
Migration status			
Residents	1	1	1
Internal migrants	1.14 (0.70–1.87)	1.26 (0.76–2.09)	1.56 (0.80–3.04)
External migrants	1.70 (1.06–2.73)	1.37 (0.80–2.33)	1.42 (0.76–2.66)
Sex			
Male	1	1	1
Female	1.31 (0.88–1.96)	1.78 (1.16–2.73)	2.37 (1.50–3.76)
Education			
Primary	1	1	1
Secondary	1.20 (0.51–2.82)	1.95 (0.57–6.72)	1.11 (0.30–4.07)
Tertiary	1.16 (0.26–5.08)	2.69 (0.56–12.90)	0.76 (0.13–4.64)
No education	0.95 (0.39–2.30)	1.55 (0.44–5.40)	0.67 (0.18–2.54)
Age			
15–25	1	1	1
25–40	2.97 (1.52–5.80)	2.10 (1.06–4.20)	2.66 (1.26–5.61)
40–59	1.84 (0.91–3.74)	1.86 (0.91–3.78)	1.63 (0.76–3.48)
Economic status			
1st Poorest	1	1	1
2nd quintile	0.85 (0.44–1.63)	0.80 (0.39–1.62)	0.75 (0.36–1.56)
3rd quintile	0.92 (0.47–1.81)	1.08 (0.54–2.17)	1.06 (0.51–2.17)
4th quintile	1.19 (0.65–2.19)	1.65 (0.89–3.06)	1.46 (0.73–2.95)
5th Least poor	0.92 (0.49–1.74)	1.21 (0.66–2.24)	1.40 (0.74–2.65)
Missing	1.42 (0.77–2.61)	1.07 (0.51–2.28)	0.49 (0.17–1.37)

### Reasons for migration

The dominant reason for migration among deceased individuals varied depending on the ART period as shown in Table 5. Prior to the introduction of ART, about 50% of diseased external migrants were reported to follow their parents as their main reason for migration into the study area. Among external migrants, 8% moved to the study area to seek for treatment compared to 2.4% of internal migrants. After widespread availability of ART, patterns of reasons for migration changed for external migrants, where the proportion of external migrants reported following parents decreased from 50 to 34%. The proportion of external migrants returning to seek for treatment decreased from 8% prior ART to 5% during ART scale-up and 1.8% after widespread availability of ART.

**Table 5:** Reasons for migration for deceased adults’ Pre-ART, ART scale up and Post ART

	Before ART		ART scale-up		Widespread of ART	
			
Reasons	Internal migrants	External migrants	Internal migrants	External migrants	Internal migrants	External migrants
Follow-parents	24.7	49.7	32.2	52.6	34	30.1
Following spouse	11.9	10.8	8.5	8.9	6.8	9.7
Change residency	33	14.4	25.4	12.6	28.2	25.7
Follow family	1.5	1.2	7.9	5.9	6.8	7.1
Married	0	0.6	4.5	3	8.7	7.1
Agriculture	2.6	2.4	7.3	7.4	5.8	7.1
Divorced	4.1	1.2	1.7	0.7	2.9	3.5
Seek treatment	2.1	7.8	1.7	5.2	1.9	1.8
Others	20.1	12	10.7	3.7	4.9	8
Total	**194**	**167**	177	135	103	113

These were the overall (both males and females).

## Discussion

This study shows higher overall mortality among external migrants compared to residents before ART became widely available in the district. Internal migrants also had higher mortality than residents before the introduction of ART. Decreasing trends in mortality rates were noted for both migrants and residents between 2000 and 2011. Sex-specific crude mortality rate for external migrants was higher for males than females. Although the overall mortality rates for females were slightly higher than those of males, there was no statistical difference in the adjusted analysis comparing male and female mortality rates in any of ART periods. Prior to ART introduction, the odds of an AIDS death was significantly higher among deaths in external migrants than in residents (AOR=1.70, 95% CI: 1.06–2.73). After widespread availability of ART, there remained higher odds of AIDS deaths among deaths in external migrants than in residents, but this was no longer significant (AOR=1.42, 95% CI: 0.76–2.66). Females were more likely to die from AIDS than males during all ART periods.

Both the crude mortality analysis and subanalysis of those who died affirm our hypothesis that scaling-up of free ART programs may possibly have contributed to the reduction of return migrants seeking care in rural areas as a result of improved health status and survival among those on ART treatment. Elevated odds of dying from AIDS for external migrants compared to residents observed in this study were comparable with findings from other cohort studies in Rural South Africa before the introduction of ART (11, 12).

The introduction of free ARV in 2004, in Tanzania, may have influenced HIV-infected migrants to stay in urban areas, who would otherwise return home for palliative care while waiting to die in the absence of treatment. This is reflected in the reduced risk of death and odds of AIDS deaths for migrants after widespread availability of ART. Treatment and care for HIV patients were made available and accessible for free to majority of health centers and hospital hence reduced migration of AIDS-infected individual to rural areas previously induced by AIDS-related illness and morbidity. High AIDS mortality rate for males external migrants compared to females could be attributed by lower ARV coverage among males as reported in routine health system in Tanzania (10, 22) and other parts of the world (24). This is due to the fact that more females than males are accessing ART services through provider-initiated programs, where all females attending antenatal clinic are counseled, tested for HIV, and those with the disease are enrolled on care or treatment (19).

Analysis of reasons for migration could be used to factor out the ‘coming home to die’ concept from other possible factors pulling individuals to rural areas. Before ART was available, more than half of the deceased external migrants reported to follow parents as their main reason for migration. However, following parents could possibly feature ‘follow parent for care or possibly die’ because the majority who reported this reason were in the age of independence (25–59 years) from their parents. This means that they may possibly return home not to stay with their parents but seek for care or treatment from their parents or relative. In many developing countries affected by the HIV epidemic, mothers are highly involved in the care of their infected sons and daughters to the point of death as well as the care of orphans left behind by their children. Majority of infected return migrants did not report return home for care or treatment as their main reason for migration in an attempt to hide their HIV. This was mainly contributed by high stigma behaviour in the study area toward HIV positive individuals and ARV users (39, 40). Men's superiority behavior observed elsewhere may also influence some of the male respondents not to report return home for care or treatment in an effort to maintain their status quo (39–41). Following widespread availability of ART, diseased individuals reporting ‘follow parents’ decreased from 50 to 34%. External migrants group was also reported to have the highest percentage of those reported returning home to seek treatment compared to internal migrants. Other reasons for migration, such as retirement, contribute little to reasons of return migrants because majority (approximately 80%) of internal and external migrants were in the age range between 25 and 59 years, while retirement age in Tanzania is 60 years and above. Since the quality of health care in urban areas is often superior to rural, then seeking care in rural could mean consulting traditional healers or waiting to die from incurable diseases such as AIDS.

This study has several limitations that need to be considered when interpreting the findings and make general conclusions to other settings. The analysis of this HDSS data mainly depended on mortality events rather than HIV prevalent cases. If HIV sero-surveys were available in the study area, we could provide more concrete analysis on return migrants and residents by HIV status. The subset analysis of those who died in the study area could be more precise in settings where the HIV status of migrants and residents was available. This analysis is restricted to individuals aged 15–59 years; therefore, care should be taken in extrapolating results to individual aged below 15 years and above 60 years. Comparability of cause of deaths over time and misclassification HIV/AIDS-related deaths should be taken into consideration. Although verbal autopsy has been successful in detecting HIV/AIDS deaths (35–37), comparability of results generated by physicians may not be consistent due to their changes in general perception of local AIDS epidemic. While some physician may perceive AIDS mortality decreasing in the population and tend to assign less frequent AIDS deaths during cause of death assignment, others may regard AIDS mortality a growing public health concern and assign AIDS death more frequent. Over the last 10 years there have been changes in physicians who assign cause of death. This could possibly result in changes in number of deaths assigned as HIV/AIDS death due to differences in implementing ICD10 procedures among physicians. TB and HIV misclassification is another limitation in this study. Co-infection of the diseases and existence of similarity in signs and symptoms may lead to misclassification (42, 43). In this study, we have taken the definition of AIDS death alone, though some other studies have grouped together the two diseases (44).

The findings in this study support the widespread evidence of benefits of ART on premature mortality and population mobility among HIV-positive migrants.

## Conclusions

This work has provided evidence that ART's universal access policy in Tanzania has averted a substantial number of deaths connected with AIDS in rural areas among internal and external migrants. Prior to ART scale-up, critically ill AIDS patients preferred to migrate to rural for care and possibly die because of the absence of affordable treatment and care. The return rural-urban migrants who are critically ill contributed in burdening the already unstable rural health-care system and families. Widespread availability of ART has reduced the burden of care to families and health-care system in rural areas. Reduction of AIDS patients seeking care in rural may have resulted in savings for families, increased time for economic activities, especially in agriculture and improving other sectors of health such maternal and newborn health.

Projection studies show 50% of African inhabitants will be living in urban area by 2,025 implying increased rural-urban migrants in the future (3). The fact that rural-urban migrants are more likely to engaging in risky sexual behaviors, health-care planners need long-term plans and investments in strengthening HIV/AIDS education both to rural and urban inhabitants. Reducing the gap in access to ARV among the poor should be the key factor in designing intervention programs aimed at optimizing the effect of ARV's to both migrants and residents. This is due to the fact that majority of rural-urban migrants are poor and their access to ARV may be compromised compared to those who are economically well in urban.

## Ethical review

The study received ethical clearance from the institutional review board of the Ifakara Health Research and Development Centre (Now Ifakara Health Institute) and the Tanzanian Medical Research Coordinating Committee.
